# Extended interval dosing of ocrelizumab modifies the repopulation of B cells without altering the clinical efficacy in multiple sclerosis

**DOI:** 10.1186/s12974-023-02900-z

**Published:** 2023-09-26

**Authors:** Carla Rodriguez-Mogeda, Zoë Y. G. J. van Lierop, Susanne M. A. van der Pol, Loet Coenen, Laura Hogenboom, Alwin Kamermans, Ernesto Rodriguez, Jack van Horssen, Zoé L. E. van Kempen, Bernard M. J. Uitdehaag, Charlotte E. Teunissen, Maarten E. Witte, Joep Killestein, Helga E. de Vries

**Affiliations:** 1 Department of Molecular Cell Biology and Immunology, Amsterdam UMC Location Vrije Universiteit Amsterdam, Amsterdam, The Netherlands; 2 Amsterdam Neuroscience, Amsterdam, The Netherlands; 3 MS Center Amsterdam, Amsterdam UMC Location Vrije Universiteit, Amsterdam, The Netherlands; 4 Department of Neurology, Amsterdam UMC Location Vrije Universiteit Amsterdam, Amsterdam, The Netherlands; 5 Department of Neurobiology and Aging, Biomedical Primate Research Centre, Rijswijk, The Netherlands; 6 Cancer Center Amsterdam, Amsterdam, The Netherlands; 7 Amsterdam Infection and Immunity Institute, Amsterdam, The Netherlands; 8 Department of Clinical Chemistry, Amsterdam UMC Location Vrije Universiteit Amsterdam, Amsterdam, The Netherlands; 9 Alzheimer Center Amsterdam, Amsterdam, The Netherlands

**Keywords:** Multiple sclerosis, B cells, Ocrelizumab

## Abstract

**Background:**

Recent studies suggest that extended interval dosing of ocrelizumab, an anti-B cell therapy, does not affect its clinical effectiveness in most patients with multiple sclerosis (MS). However, it remains to be established whether certain B cell subsets are differentially repopulated after different dosing intervals and whether these subsets relate to clinical efficacy.

**Methods:**

We performed high-dimensional single-cell characterization of the peripheral immune landscape of patients with MS after standard (SID; n = 43) or extended interval dosing (EID; n = 37) of ocrelizumab and in non-ocrelizumab-treated (control group, CG; n = 28) patients with MS, using mass cytometry by time of flight (CyTOF).

**Results:**

The first B cells that repopulate after both ocrelizumab dosing schemes were immature, transitional and regulatory CD1d^+^ CD5^+^ B cells. In addition, we observed a higher percentage of transitional, naïve and regulatory B cells after EID in comparison with SID, but not of memory B cells or plasmablasts. The majority of repopulated B cell subsets showed an increased migratory phenotype, characterized by higher expression of CD49d, CD11a, CD54 and CD162. Interestingly, after EID, repopulated B cells expressed increased CD20 levels compared to B cells in CG and after SID, which was associated with a delayed repopulation of B cells after a subsequent ocrelizumab infusion. Finally, the number of/changes in B cell subsets after both dosing schemes did not correlate with any relapses nor progression of the disease.

**Conclusions:**

Taken together, our data highlight that extending the dosing interval of ocrelizumab does not lead to increased repopulation of effector B cells. We show that the increase of CD20 expression on B cell subsets in EID might lead to longer depletion or less repopulation of B cells after the next infusion of ocrelizumab. Lastly, even though extending the ocrelizumab interval dosing alters B cell repopulation, it does not affect the clinical efficacy of ocrelizumab in our cohort of patients with MS.

**Supplementary Information:**

The online version contains supplementary material available at 10.1186/s12974-023-02900-z.

## Background

Multiple sclerosis (MS) is an autoimmune, demyelinating disease of the central nervous system (CNS). Historically, patients with MS are distinguished by different clinical categories: in the majority of patients, the disease starts with exacerbations of neurological symptoms followed by periods of remission—called relapsing–remitting MS (RRMS). Often these patients develop secondary progressive MS (SPMS), where disability accrues over time. A small percentage of patients acquire primary progressive MS (PPMS), which is characterized by disability worsening from disease onset [1]. However, more recent evidence indicates that both relapse activity and progression independent from relapses occur throughout the disease, albeit at varying degrees [2, 3]. To date, extensive evidence points towards a crucial role of B cells in the pathophysiology of MS and hence, multiple therapies that target B cells are currently used in daily clinics [4].

Ocrelizumab is a humanized monoclonal antibody against CD20, which selectively depletes CD20^+^ B cells via multiple mechanisms: apoptosis, antibody-dependent cell-mediated and complement-dependent cytotoxicity, and antibody-dependent cellular phagocytosis [5]. Ocrelizumab is now widely used for the treatment of RRMS patients and it effectively reduces relapse rates [6]. Furthermore, it is the first approved therapy for PPMS and delays disability progression in a subset of these patients that have radiological activity [7].

As the efficacy of ocrelizumab depends on the consistent depletion of peripheral B cells, specifically memory B cells, treatment with ocrelizumab had significant implications during the COVID-19 pandemic [8, 9]. On one hand, continuous depletion of B cells increases the risk of severe respiratory infections since less than one in five ocrelizumab-treated patients with MS developed an antibody response against COVID-19 [10]. On the other hand, patients needed to postpone or delay ocrelizumab treatment due to COVID-19 infection and/or worsening of the epidemiological situation in the hospital or due to safety concerns, which might impact the effectiveness of the treatment. Accordingly, international MS experts suggested extending the interval between ocrelizumab doses, while following patients’ clinical status [11–13].

This study was undertaken to determine how extended dosing of ocrelizumab modifies the repopulation of specific B cell subsets and if these cell subsets affected the effectiveness of the treatment. For this, we used single-cell mass cytometry by time of flight (CyTOF) to profile the circulating immune cells after standard dosing or extended dosing of ocrelizumab and we compared findings to patients with MS that were not-treated with ocrelizumab. Finally, we studied how B cell subsets change over time with different dosing schemes within the same patients.

## Methods

### Study population

Patients with MS were recruited for personalized dosing of ocrelizumab between 15 March 2020 and 1 November 2020 [11]. All subjects provided written informed consent to use medical data and materials for scientific research. This study was approved by the institutional ethics committee (Amsterdam UMC Ethics Committee).

In summary, blood was drawn starting 24–30 weeks after the dose of ocrelizumab (600 mg or 2 × 300 mg in two consecutive weeks). With this blood, CD19^+^ B cells were counted using flow cytometry to decide whether a next ocrelizumab dose was needed two weeks later. If the B cell count was below 10 cells/µL, the next ocrelizumab dose was withheld for another four weeks. Follow-up B cell counts were repeated every 4 weeks and re-dosing happened when CD19^+^ B cells increased above the cut-off of 10 cells/μL [11]. Accordingly, and based on expert opinion [11], we defined the intervals of personalized dosing as standard interval dosing (SID) when the next dose of ocrelizumab was scheduled as a regular maintenance interval, less than 30 weeks since the previous dose; and extended interval dosing (EID), when the dose of ocrelizumab was scheduled with a delay, i.e. more than 30 weeks after the previous dose. Two weeks after CD19^+^ B cell counts reached 10 cells/μL, a new blood sample was taken and stored for further use in this study. All these blood samples were taken just before the next dosage of ocrelizumab and consequently, they were used to study B cell repopulation. We also included patients with MS that did not receive ocrelizumab as control group (CG). These patients were treated with other disease modifying therapies (DMTs) but were planned to switch to ocrelizumab (glatiramer acetate, n = 5; dimethyl fumarate, n = 10; natalizumab, n = 4; teriflunomide, n = 2; fingolimod, n = 2; IFN, n = 2; alemtuzumab, n = 1; untreated, n = 2). Crucially, the blood sample taken for the patient treated with alemtuzumab was more than three months after the infusion of alemtuzumab. Patients underwent neurological examination close to blood sampling, including determination of the expanded disability status scale (EDSS) and relapse assessment, annual brain magnetic resonance imaging (MRI) to monitor radiological disease activity (T2 lesions and/or gadolinium-enhancing lesions), and measurement of serum neurofilament light (sNfL) with Simoa Advantage Kit (Quanterix) to assess neuronal damage [11]. Demographic and clinical characteristics of the participants are provided in Table 1. For a subset of patients, we had access to a second blood sample after a subsequent ocrelizumab infusion, which we included to study this cohort longitudinally.

**Table 1:** Patient characteristics and disease parameters of the cohort

	All	CG	SID	EID
Individuals	108	28	43	37
Sex (female)	65 (60.18%)	22 (78.57%)	27 (62.79%)	16 (43.24)
Age (mean ± SD)	41.67 ± 11.42	38.89 ± 11.54	41.69 ± 11.93	43.72 ± 10.54
Type of MS				
RRMS	84 (77.77%)	23 (82.14%)	35 (81.39%)	26 (70.27%)
SPMS	4 (3.70%)	0	3 (6.97%)	1 (2.70%)
PPMS	19 (17.59%)	4 (14.28%)	5 (11.62)	10 (27.02%)
Disease duration (mean ± SD)	10.13 ± 6.95	8.19 ± 6.80	10.79 ± 7.48	10.82 ± 6.29
EDSS^a^ (mean ± SD)	4.09 ± 1.75	3.93 ± 1.74	3.75 ± 1.70	4.39 ± 1.81
Serum NfL^b^ (pg/mL, mean ± SD)	8.51 ± 3.61	9.35 ± 3.13	8.41 ± 4.38	8.44 ± 3.51
Radiological activity^c^ (mean ± SD)	0.76 ± 2.00	2.72 ± 3.28	0.17 ± 0.67*	0.08 ± 0.36*
Number of relapses (mean ± SD)	0.1 ± 0.30	0.32 ± 0.48	0.04 ± 0.22*	0 ± 0*
BMI (mean ± SD)	23.75 ± 3.35	26.65 ± 5.88	24.25 ± 3.39	22.80 ± 2.97
Number of prior OCR infusions (mean ± SD)	NA	NA	3.26 ± 1.03	3.97 ± 1.24
Patients with a follow-up sample:				
SID		7	23	26
EID		4	19	10

^a^Expanded disability status scale

^b^Neurofilament light

^c^Number of new lesions close to sampling (± 2 months)

*Significant differences compared to CG

### Immune cell isolation

Peripheral blood mononuclear cells (PBMCs) were isolated in Vacutainer Mononuclear Cell Preparation Tubes with sodium citrate (CPT, BD Biosciences) according to the manufacturer’s instructions. PBMCs were stored at a concentration of 5 × 10^6^ cells/mL in liquid nitrogen until further analysis.

### CyTOF antibody labelling and titration

Antibody labelling with the indicated lanthanide metal tag was performed using the MaxPar antibody conjugation kit (Standard BioTools) according to the manufacturer’s protocol. Briefly, 100 µg of antibody was washed with R-buffer using a 50 kDa Eppendorf filter (Millipore) and 100 μL of 4 mM tris(2-carboxyethyl)phosphine (TCEP)-R-buffer was added and incubated at 37 °C for 30 min. Filters were then washed with R-buffer followed by a C-buffer wash. Concurrently, 5 μL of lanthanide metal solution was added to a mix of polymer and l-buffer and incubated at 37 °C for 30–40 min. The lanthanide-loaded polymer was washed in a 3 kDa Eppendorf filter (Millipore), first with l-buffer and then with C-wash. Afterwards, the lanthanide-loaded polymer was mixed with the partially reduced antibody in the 50 kDa filter and incubated at 37 °C for 90 min. The labelled antibody was collected by inverting the 50 kDa filter over a new collection tube and centrifuging at 1000*g* for 2 min. The antibody concentration was determined using the Implen™ NanoPhotometer N60 and diluted to 0.5 mg/mL using an equal amount of MaxPar PBS and antibody stabilizer buffer (Candor Biosciences). Antibodies were stored at 4 °C until further use. All antibodies used in this study were titrated using unfixed thawed PBMCs and the most optimal concentration with the least spillover was chosen.

### Generation of the CyTOF reference sample

We generated a reference sample to use in each CyTOF run to adjust for batch effects, including differences in staining intensity between batches due to technical variations in the protocol or daily changes in instrument functioning. This reference sample contained PBMCs from 2 healthy controls of which half of the PBMCs were divided into equal amounts and stimulated with cytokines (IL-1b (10 ng/mL, PeproTech), IL-4 (10 ng/mL, ImmunoTools), LPS (10 ng/mL, Sigma), TGFb1 (100 ng/mL, Miltenyi), Dexamethasone (100 nM, Sigma), TNF (10ng/mL, PeproTech), IFN (10 ng/mL, PeproTech), PolyIC (500 ng/mL, Amersham Pharmacia Biotech, NJ, USA) and a mix of PMA (25 ng/mL, Sigma) and ionomycin (100 ng/mL, Sigma)) at 37 °C for 12 h to induce expression of all markers that were included in the CyTOF panel. For one of the donors, we isolated B cells following the manufacturer’s guidelines (EasySep Human B cell enrichment kit, Stem cell) to spike the reference sample to include more B cells. Unstimulated PBMCs, cytokine-stimulated PBMCs and isolated B cells were combined and stored in aliquots at –70 °C until further use.

### CyTOF staining protocol and freezing of immune cells

PBMCs were thawed rapidly and transferred to a 15 mL tube with 1mL of fetal calf serum (FCS; Corning). Samples were washed twice with RP10 (RPMI 1640, 10% FCS, 1% penicillin/streptomycin, 1% glutamine), resuspended in MaxPar PBS (Standard BioTools) and transferred to a 96-V-bottom plate. Between washes, cells were counted. For each patient and reference sample, 2 × 10^6^ cells were stained for 7.5 min at 37 °C with the viability marker Cell-ID Cisplatin-198Pt (1:1000, diluted in MaxPar PBS; Standard BioTools). FCS was added to quench cisplatin followed-up by a wash in cell staining buffer (CSB; Standard BioTools). Cells were then incubated with Human TruStain FcX Fc receptor blocking solution (1:50, diluted in CSB) together with CD49-89Y. After 10 min, the appropriate CD45 barcoding mixes (Standard BioTools) were added to the corresponding samples and incubated for 30 min more (Table 2). We included 32 patient samples and 2 reference samples for each batch of staining and running. After centrifugation, samples were washed twice in CSB and the cells from all samples were pooled. Surface antibody cocktail (Table 2) was prepared fresh in CSB and added to the combined cell mix in a cell-to-antibody cocktail ratio of 3 × 10^6^ cells/100 µL. After a 30-min incubation, cells were washed twice with CSB and once with MaxPar PBS. Then, cells were fixated with 1.6% fresh paraformaldehyde (PFA, Thermo Fisher) in MaxPar PBS for 15 min. After centrifugation, cells were permeabilized with a 30-min incubation in FoxP3 Fix/Perm working solution (eBioscience), followed by two washes with 1 × Permeabilization buffer (eBioscience). Antibody cocktail containing intra-nuclear markers was prepared in 1 × Permeabilization buffer and added to the cells in the same cell-to-antibody cocktail ratio (3 × 10^6^ cells/100 µL; Table 2). Cells were incubated for 45 min, then washed three times with 1 × Permeabilization buffer and fixated with 1.6% fresh PFA in MaxPar PBS for 15 min. After centrifugation, fixed cells were stained with MaxPar Intercalator-Ir (1:4000 diluted in MaxPar Fix and Perm buffer; all Standard BioTools) for 1 h. Cells were counted and divided over approximately 5 × 10^6^ cells/cryovial, and each cryovial was filled up to 1–1.5 mL with MaxPar Intercalator-Ir solution. Cells were frozen at –70 °C until sample acquisition. During the CyTOF staining, all reagents were kept on ice, and centrifugation steps were performed at 1500 rpm before and 2000 rpm after fixation for 7 min at 4 °C (acceleration 9 and deceleration 7). All incubations were performed at room temperature in a shaker unless stated otherwise.

**Table 2:** Antibodies for CyTOF

Metal	Antigen	Clone	Manufacturer	Concentration (µL) per 100 µL	Cocktail
106Cd	CD45	HI30	Standard BioTools	0.25	Barcoding
110Cd	CD45	HI30	Standard BioTools	0.25	Barcoding
111Cd	CD45	HI30	Standard BioTools	0.25	Barcoding
112Cd	CD45	HI30	Standard BioTools	0.25	Barcoding
113Cd	CD45	HI30	Standard BioTools	0.25	Barcoding
114Cd	CD45	HI30	Standard BioTools	0.25	Barcoding
116Cd	CD45	HI30	Standard BioTools	0.25	Barcoding
89Y	CD45	HI30	Standard BioTools	1	Surface
141Pr	CD235ab	HIR2	Standard BioTools	0.5	Surface
142Nd	CD11a	HI111	Standard BioTools	0.5	Surface
143Nd	CD5	UCHT2	Standard BioTools	1	Surface
144Nd	CD69	FN50	Standard BioTools	0.5	Surface
145Nd	CD16	3G8	Standard BioTools	0.25	Surface
146Nd	IgD	IA6-2	Standard BioTools	0.25	Surface
147Sm	CD20	2H7	Standard BioTools	0.5	Surface
148Nd	IgA	Polyclonal	Standard BioTools	0.5	Surface
149Sm	CD25 (IL-2R)	2A3	Standard BioTools	1	Surface
150Nd	CD138	DL-101	Standard BioTools	0.5	Surface
151Eu	CD14	M5E2	BioLegend	1	Surface
152Sm	CD21	BL13	Standard BioTools	0.5	Surface
153Eu	CD8a	RPA-T8	Standard BioTools	0.0625	Surface
154Sm	CD3	UCHT1	Standard BioTools	0.25	Surface
155Gd	CD45RA	HI100	Standard BioTools	0.25	Surface
156Gd	CD86	IT2.2	Standard BioTools	0.25	Surface
158Gd	CD1d	51.1	BioLegend	1	Surface
159Tb	CD11c	Bu15	Standard BioTools	0.125	Surface
161Dy	PD-1	EH12.2H7	BioLegend	0.375	Surface
163Dy	CD56 (NCAM)	NCAM16.2	Standard BioTools	0.015625	Surface
164Dy	CD166	3A6	Standard BioTools	0.5	Surface
165Ho	CD19	HIB19	Standard BioTools	0.25	Surface
166Er	CD24	ML5	Standard BioTools	1	Surface
167Er	CD38	HIT2	Standard BioTools	0.5	Surface
168Er	CD22	HIB22	Standard BioTools	0.25	Surface
169Tm	CD57	HNK-1	BioLegend	0.125	Surface
170Er	CD54	HA58	Standard BioTools	0.125	Surface
171Yb	CD162	KPL-1	BioLegend	0.125	Surface
172Yb	IgM	MHM-88	Standard BioTools	0.125	Surface
173Yb	HLA-DR	L243	Standard BioTools	0.5	Surface
174Yb	CD49d	9F10	Standard BioTools	0.125	Surface
175Lu	CD27	O323	BioLegend	0.25	Surface
176Yb	CD4	RPA-T4	Standard BioTools	0.25	Surface
209Bi	CD11b (Mac-1)	ICRF44	Standard BioTools	0.5	Surface
160Gd	Tbet	4B10	Standard BioTools	0.75	Nuclear
162Dy	FoxP3	PCH101	Standard BioTools	1	Nuclear

### CyTOF sample acquisition

Each cryovial was defrosted and washed twice with CSB. Cells were divided into two falcon tubes (approximately 1.5 × 10^6^ cells/tube). One tube was kept at 4 °C with the cells as pellet and the second tube was washed with cell acquisition solution (CAS; Standard BioTools). Cells in CAS solution were diluted to 300–600 cells/0.5 μL and filtered into a new tube. Calibration beads were added to the cell suspension to 15% of the final volume. Cells were acquired on a Helios™ (Standard BioTools) with an event rate of 300–400 events per second. Runs took approximately 45 min. Each cryovial was used for 2–4 runs. During the day, tuning of the machine was performed during start-up and after two consecutive runs. After each run, wash solution was run for 5 min, followed by a small dip of the sample introduction in milliQ water and a CAS run for 10 min.

### CyTOF data pre-processing

Acquired samples were randomized using Gaussian negative half-zero randomization in CyTOF software version 6.7. The obtained FCS files were normalized using bead normalization and concatenated using the CyTOF software version 6.7. The barcoded FCS files were uploaded into OMIQ analysis software (https://www.omiq.ai/) and beads, cell debris and cell doublets were removed from the data of each barcoded FCS file using DNA, beads and Gaussian parameters (Additional file 1: Fig. S1A) [14]. Next, CD45^+^ cells were selected for follow-up pre-processing and all channels were inspected carefully. At the end of the runs, we saw an upward drift in the background levels of CD5, CD27 and T-bet. We used the *FlowCut* package [15] in OMIQ to remove outlier events over time resulting from clogs or increased background signal. Then, FCS files were imported into Rstudio (version 4.2.1) for debarcoding using *CATALYST* [16]. Debarcoded FCS files were uploaded into OMIQ and live cells were selected using negative reactivity for Cell-ID Cisplatin-195Pt, negative reactivity for erythroid marker CD235a-141Pr and positive reactivity for CD45-89Y. Finally, batch alignment and normalization based on the reference samples were performed using CytoNorm in OMIQ [17]. Processed FCS files including live immune cells were used for further processing steps.

### CyTOF data processing

Data analysis of the processed FCS files was performed in OMIQ. We selected T cells (CD3^+^ CD19^−^), B cells (CD3^−^ CD19^+^ CD11b^−^) and the “rest” of cells (CD3^−^ CD19^−^ CD11b^+^) following the gating strategy shown in Additional file 1: Fig. S1B. We furthermore annotated the number of T cells, B cells and the rest of cells per sample. Due to computational power capabilities in OMIQ, for downstream analysis, we subsampled a maximum of 10,000 cells/sample for the general PBMC analysis, 15,000 T cells/sample for the T cell analysis, 50,000 B cells/sample for the B cell analysis and, 15,000 “rest” cells/sample for the rest of cells. Data were visualized using Uniform Manifold Approximation and Projection (UMAP, [18]) and cells were appointed to each cluster by Phenotyping by Accelerated Refined Community (PARC, [19]). We manually merged clusters and biologically annotated relevant immune cell subsets by visual inspection of the PARC-derived clusters on the UMAPs and the clustered heatmaps that compare median marker expression between clusters. Counts per immune cell subset were then exported and their fraction of the total amount of immune cells per individual was calculated. Importantly, when calculating this fraction, we multiplied the fraction of each immune cell subset in the subsampled sample per the fraction of the correspondent cell annotated numbers of T cells, B cells and the rest of cells divided by the total number of cells imputed per individual. In the case of B cell subsets, we also calculated the fraction of each B cell subset within the amount of all B cells. Median marker expression was also exported for each subset.

### Statistical analysis

We applied different statistical models to analyse the percentage of immune cell subsets and median marker expression per subset in either the cross-sectional or the longitudinal study. In more detail, for the cross-sectional cohort, outliers were detected first using Grubb’s test. In Rstudio (version 4.2.1), fractions of the immune cells were asinh transformed to generate a normal distribution. Using the function *glm*, a multivariate generalized linear model with Gaussian distribution was applied. The fractions of cell subsets per sample were used as response variable, the treatments/interval of dosing as explanatory variable and age, sex and type of MS as covariates. Since the data obtained is highly dependent (for example, if the percentage of one subset among all immune cells goes up, it means that another subset is going down), we adjusted p-values using the Benjamini and Yekutieli method (BY) [20]. To study the median marker expression between patient groups, we used the function *lm* to apply a linear model with age, sex and type of MS as covariates. We adjusted the p-values with the Benjamini–Hochberg method [21]. After each model, we confirmed normal distribution by inspecting the distribution of the residuals in a QQ-plot and/or applying Shapiro–Wilk test. For the longitudinal cohort, we applied a similar approach but we used the function *lmer* to build a linear mixed model where we also included the patient ID as mixed effects to control for patient heterogeneity over time. To longitudinally study the change of percentages between groups of patients, we calculated the change or delta (∆) by subtracting the ratio of the immune cell subset at the second time point minus the ratio at the first time point. We further generated a linear model, as similarly done with the cross-sectional cohort, where the change of ratio or delta was used as response variable. Data were judged to be statistically significant when adjusted p-value < 0.05. Quantitative data are shown as independent data points in plots made in Graphpad Prism (version 9) or Rstudio using *ggplot2*. In the case of CD3^+^CD19^−^ and CD3^−^ CD19^−^ populations, we only display plots with statistically significant differences when p-value < 0.05 or when adjusted p-value < 0.05. For the immune network analysis, we performed Spearman correlations of the percentage of all immune cell subsets and we adjusted the p-value using BY method in Rstudio. We only plot the correlations that had a Rho value > 0.5 or < − 0.5 and that were statistically significant after p-value adjustment. Of note, we also only show the correlations that involve the immune cell subsets found in the CyTOF and not their parents (e.g. correlations for naïve CD4^+^ T cells, and not for CD4^+^ T cells). We similarly performed Spearman correlations of the percentage of immune cell subsets with the patient characteristics and clinical data. We only performed Spearman correlations when there were more than 10 data points included. In this case, we display all Rho values and we indicate statistically significant differences when p-value < 0.05 or when adjusted p-value < 0.05.

## Results

### Ocrelizumab predominantly affects B cells

**Fig. 1:**
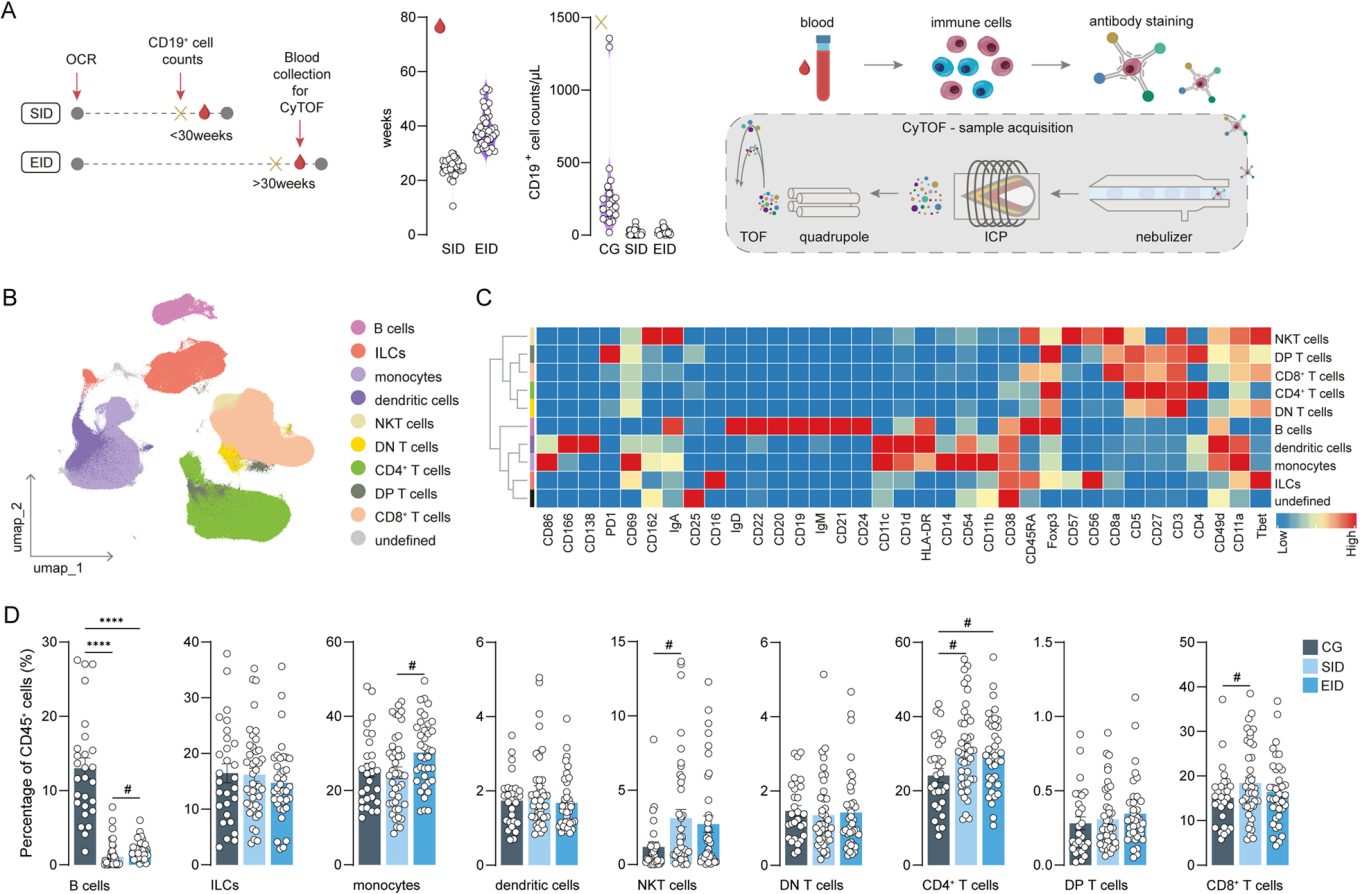
Immunophenotyping after SID and EID on total PBMCs shows repopulation of B cells. **A** Schematic overview of the study design and timeline. Violin plots display the number of weeks after the first infusion of ocrelizumab and the B cell counts, which allow us to divide patients with MS into SID or EID. PBMCs from patients were collected before the next ocrelizumab infusion and mass cytometry by time-of-flight (CyTOF) was performed. **B** UMAP plot showing CD45^+^ cells from the blood of CG, SID and EID patients. Colours correspond to PARC-guided clustering. Unsupervised clustering allows discrimination of the main immune cell populations. **C** Heatmap displaying the median scaled intensities of all the markers across the annotated immune cell clusters. **D** Bar plots of the percentage of each annotated cell population out of the total CD45^+^ cells from CG, SID and EID patients. Each data point corresponds to each individual, columns and error bars show mean ± SEM. P-values indicate the statistical differences after a GLM model with age, sex and type of MS as covariates. *adjusted p-value < 0.05, #unadjusted p-value < 0.05. *OCR* ocrelizumab, *PBMCs* peripheral blood mononuclear cells, *ILCs* innate lymphocyte cells, *NKT* natural killer T cells, *DN* double negative, *DP* double positive cells, *GLM* multivariate general linear model, *CG* control group, *SID* standard interval dosing, *EID* extended interval dosing

In our cohort, we included patients with standard interval dosing (SID; mean: 24.9 weeks; n = 43), extended interval dosing (EID; mean: 39.2 weeks, n = 37) of ocrelizumab, and non-ocrelizumab treated patients as controls (control group, CG; n = 28) (Fig. 1A; Table 1). As previously mentioned, CD19^+^ B cell counts were done to assess the repopulation of B cells and two weeks after that, blood samples for immune cell collection were taken just before the ocrelizumab infusion (Fig. 1A). In this case, no differences in total CD19^+^ B cell counts were observed between SID and EID patients.

To investigate whether extended or standard dosing of ocrelizumab affects B cell repopulation, we performed mass cytometry by time of flight (CyTOF) using a 38-antibody panel designed to detect the main immune cell populations, B cell subsets and CNS migratory markers (Fig. 1A). We performed unsupervised clustering of the PBMCs of CG, SID and EID patients and by looking at the median expression of each of the markers, we could biologically merge and annotate the clusters based on biological knowledge (Fig. 1B, C; Additional file 2: Table S1). As expected, the most striking change was a decreased percentage of total B cells among all the immune cells in SID and EID patients compared to CG patients (Fig. 1D; Additional file 2: Table S2). Interestingly, we observed a slight increase of B cells in EID compared to SID despite equal total B cell counts two weeks before (Fig. 1A), which indicates that more B cells have repopulated in those two weeks after EID than after SID. In addition, we found NKT, CD4^+^ and CD8^+^ T cells to be upregulated in one or both of the ocrelizumab-treatment groups, although these numbers were only statistically significant before p-value adjustment. Similarly, a slightly higher percentage of monocytes in EID versus SID patients was found. Altogether, after ocrelizumab treatment, B cells are depleted, and consequently, the percentage of certain other immune cells, such as NKT, CD4^+^, CD8^+^ T cells and monocytes, are modestly increased. Finally, there is a trend towards more B cell repopulation in EID versus SID.

### Extended dosage of ocrelizumab leads to increased repopulation of immature and transitional B cell subsets

**Fig. 2:**
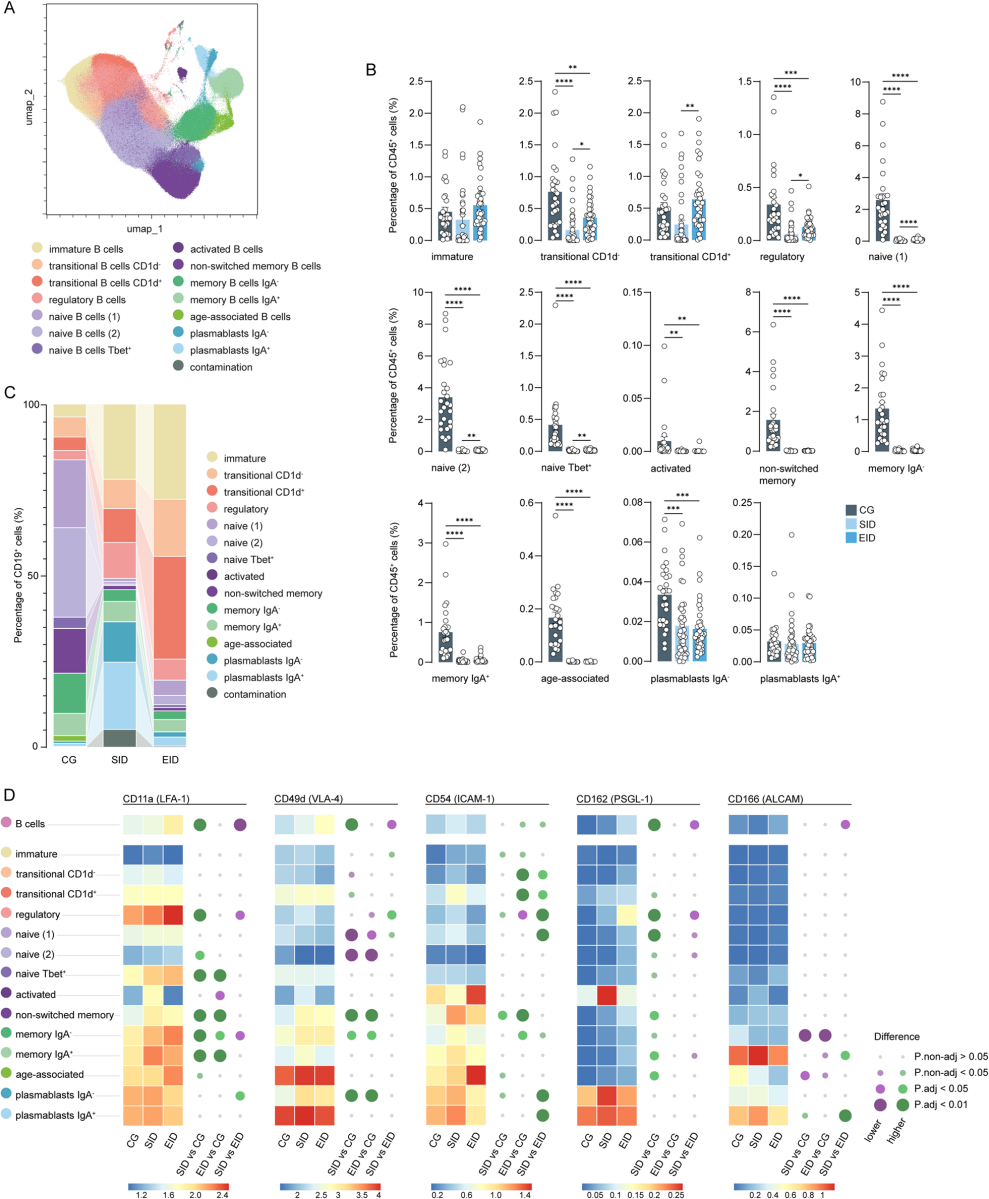
Immature, transitional and regulatory B cells are the first B cells to repopulate after SID and EID. **A** UMAP plot displays pre-gated CD19 + cells from the blood of CG, SID and EID patients. Colours correspond to PARC-guided clustering. **B** Percentage of each annotated B cell population out of the total CD45^+^ cells from CG, SID and EID patients. Each data point corresponds to each individual, columns and error bars show mean ± SEM. P-values indicate the statistical differences after a GLM model with age, sex and type of MS as covariates. **C** Proportional stacked bar graph showing the percentage of each annotated B cell cluster out of the CD19^+^ cells from CG, SID and EID patients. **D** Heatmap indicates the median scaled intensity of the corresponding markers across B cell subpopulations. The size of the circles (right) represents the value of the p-value and the colour represents the direction of change between the different experimental groups. *adjusted p-value < 0.05, **adjusted p-value < 0.01, ***adjusted p-value < 0.001, ****adjusted p-value < 0.0001. *GLM* multivariate general linear model, *CG* control group, *SID* standard interval dosing, *EID* extended interval dosing

Using our CyTOF antibody panel, we identified 14 different B cell subsets, corresponding to all stages of B cell development and differentiation (Fig. 2A; Additional file 1: Fig. S2A, Additional file 2: Table S1). In both SID and EID patients, we observed substantial repopulation of immature and transitional CD1d^−^ and CD1d^+^ B cell subsets, with a significant increase in repopulation of both transitional B cell subsets in EID patients compared to SID patients. A similarly increased percentage in EID was seen for all naïve and regulatory B cell subsets, although much lower than at CG (Fig. 2B). Overall, there was a decrease of all B cell subsets after ocrelizumab compared to CG patients, except for immature, transitional CD1d^+^, and plasmablasts IgA^+^ B cells (Fig. 2b), which indicates that these cells are the first to repopulate or not depleted by ocrelizumab. All data and corresponding unadjusted and adjusted p-values can be found in Additional file 2: Table S2.

To study the composition of the B cell compartment, we next assessed the percentage of B cell subsets in the total CD19^+^ B cell population. Within all CD19^+^ B cells, EID patients had higher percentages of immature, transitional and naïve B cells than SID patients (Fig. 2C; Additional file 1: Fig. S2B, Additional file 2: Table S3), whereas plasmablasts made up a significantly larger fraction of the B cells in SID. In line with the previous results, this suggested that EID patients have more B cell repopulation, indicated by the increase of more early B cells such as naïve and transitional B cells. As a result, the percentage of plasmablasts within all CD19^+^ B cells is higher in SID than in EID (Fig. 2C; Additional file 1: Fig. S2B), but within all CD45^+^ immune cells, is not (Fig. 2A).

### Repopulated B cells have increased migratory marker expression

We further investigated the expression of the migratory markers CD11a (LFA-1), CD49d (VLA-4), CD54 (ICAM-1), CD162 (PSGL-2) and CD166 (ALCAM), which are involved in B cell migration across CNS barriers [22, 23], on the different B cell subsets. Interestingly, when looking at all B cells together, we found a significant increase of CD11a, CD49d and CD162 in SID patients compared to CG, and a trend towards increased CD54 in EID patients compared to CG (Fig. 2D; Additional file 2: Table S4). Especially memory B cells in the SID group displayed increased expression of CD11a, CD54 and CD162 compared to CG patients. Similar trends were observed in EID patients compared to CG. However, this was not the case for naïve B cells expressing CD49d, which was lower expressed in SID and EID patients than in CG. Furthermore, we found low expression of CD166 in the majority of B cell subsets and could not find differences between treated or non-treated ocrelizumab patients. Only memory B cells and plasmablasts had high expression of CD166 and it was mostly reduced in SID or EID patients compared to CG patients. Lastly, we observed a lower expression of CD11a, CD49a, CD162 and CD166 in SID in all B cells compared to EID. Taken together, our data show that repopulated B cells in both SID and EID patients, and in particular memory B cells, have either increased activity or migratory capacity to cross the CNS barriers.

### Ocrelizumab treatment affects to a lesser extent myeloid, T and NK cells

**Fig. 3:**
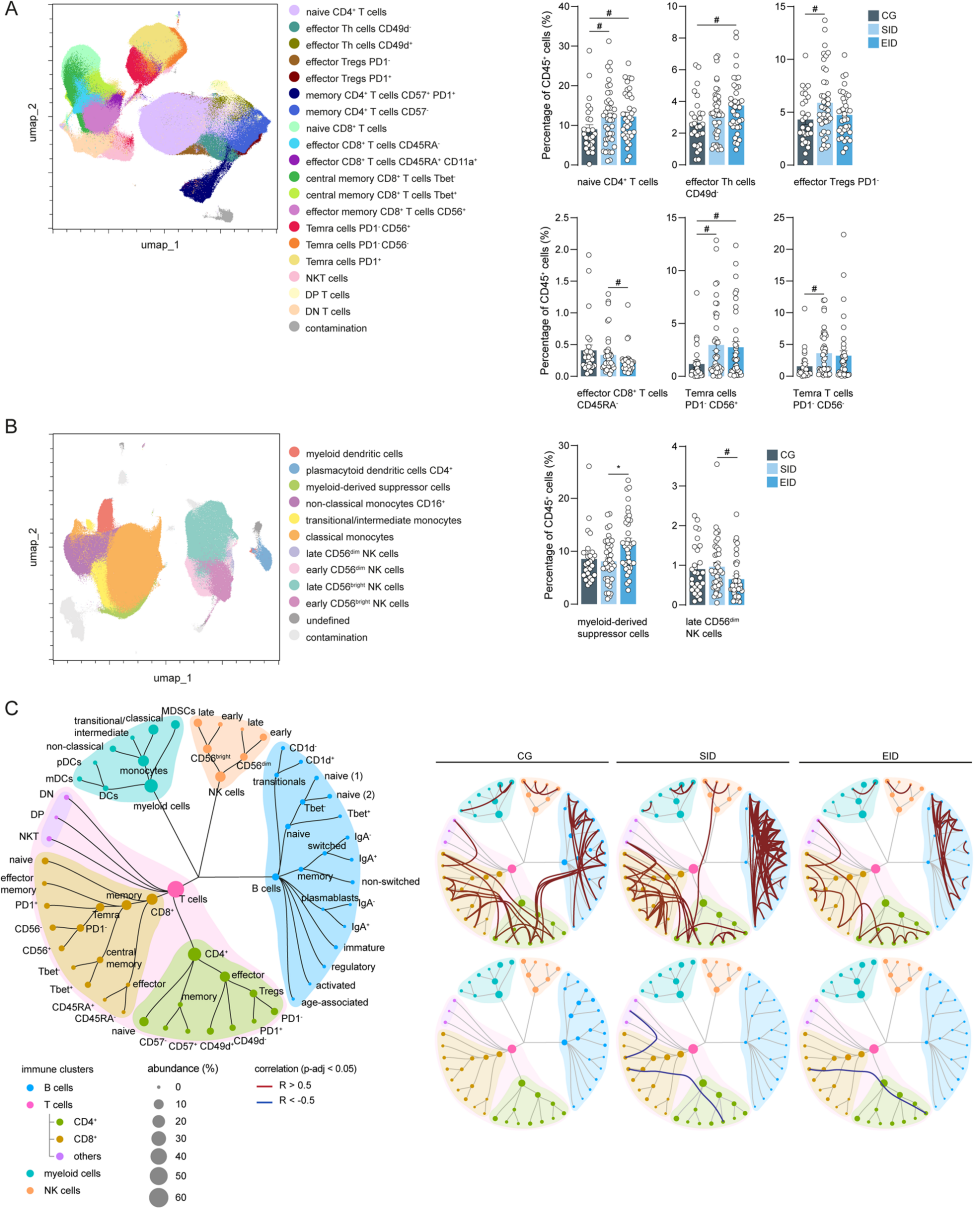
Effect of ocrelizumab in CD19^−^CD3^+^ and CD19^−^CD3^−^ cells. **A** UMAP plot displays pre-gated CD3^+^ cells from the blood of CG, SID and EID patients. Colours correspond to PARC-guided clustering. Percentage of annotated T cell populations out of the total CD45^+^ cells from CG, SID and EID patients. **B** UMAP shows pre-gated CD19^−^CD3^−^ cells from the blood of CG, SID and EID patients. Colours correspond to PARC-guided clustering. Percentage of annotated CD19^−^CD3^−^ cell clusters out of the total CD45^+^ cells from CG, SID and EID patients. **C** Spearman correlation immune network with nodes visualizing immune subsets and lines displaying correlation coefficients between immune cell clusters. The size of the node represents the abundance of the immune cell subset and the colour nodes represent the immune type. Lines represent significant positive (red) and negative (blue) correlations between clusters. **A**, **B** Each data point corresponds to each individual, columns and error bars show mean ± SEM. P-values indicate the statistical differences after a GLM model with age, sex and type of MS as covariates. *adjusted p-value < 0.05, #unadjusted p-value < 0.05. *Th* T helper cells, *Tregs* regulatory T cells, *Temra* T effector memory re-expressing CD45RA cells, *NKT* natural killer T cells, *DN* double negative, *DP* double positive cells, *NK* natural killer cells, *MDSCs* myeloid-derived suppressor cells, *pDCs* plasmacytoid dendritic cells, *mDCs* myeloid dendritic cells, *GLM* multivariate general linear model, *CG* control group, *SID* standard interval dosing, *EID* extended interval dosing

We then proceeded to further subcluster T cells and the rest of immune cells (Additional file 1: Fig. S1B). In the case of T cells, we defined 19 different subsets, ranging from naïve to effector to memory CD4^+^ and CD8^+^ T cells based on well-established markers (Fig. 3A; Additional file 1: Fig. S3A, Additional file 2: Table S1). Overall, we observed a small increase after ocrelizumab treatment in both dosing groups of naïve CD4^+^ T cells, effector Th cells CD49d^−^, effector Tregs PD1^−^ and Temra cells PD1^−^ CD56^+^ and PD1^−^ CD56^−^, albeit non-significant after p-value adjustment. Only for effector CD8^+^ T cells CD45RA^−^ there were differences between dosing intervals of ocrelizumab, with a small decrease after EID compared to SID, although this difference was also not significant after adjustment (Fig. 3A). Within CD3^−^CD19^−^ immune cells, we detected 11 different populations, including subsets of dendritic cells, monocytes and NK cells (Fig. 3B; Additional file 1: Fig. S3B, Additional file 2: Table S1). While the percentages of most immune cell subsets were similar between non-treated patients with ocrelizumab and the two ocrelizumab-treated patient groups, we observed a significant increase of myeloid-derived suppressor cells and a trend towards decreased late CD56^dim^ NK cells in EID patients compared to SID patients (Fig. 3B). All the data for other immune cell presence and corresponding unadjusted and adjusted p-values can be found in Additional file 2: Table S2. Next, we assessed changes in the immune connectome after ocrelizumab treatment. Overall, we did not detect many correlations across subsets of the main immune cell types, but observed many positive correlations between subsets within the various immune cell types, especially in T and B cells. Interestingly, in the CG group, memory B cells correlated positively with effector CD4^+^ T cell subsets, however, these correlations were lost in SID and EID patients. Of note, correlations within T cells, myeloid cells and NK cells were very similar in CG and patients with both dosing schemes of ocrelizumab, indicating that ocrelizumab has a very limited effect on other immune cells besides B cells (Fig. 3C).

### Repopulated B cell subsets express higher levels of CD20 after extended dosing, thereby increasing the effectiveness of consecutive ocrelizumab dosing

**Fig. 4:**
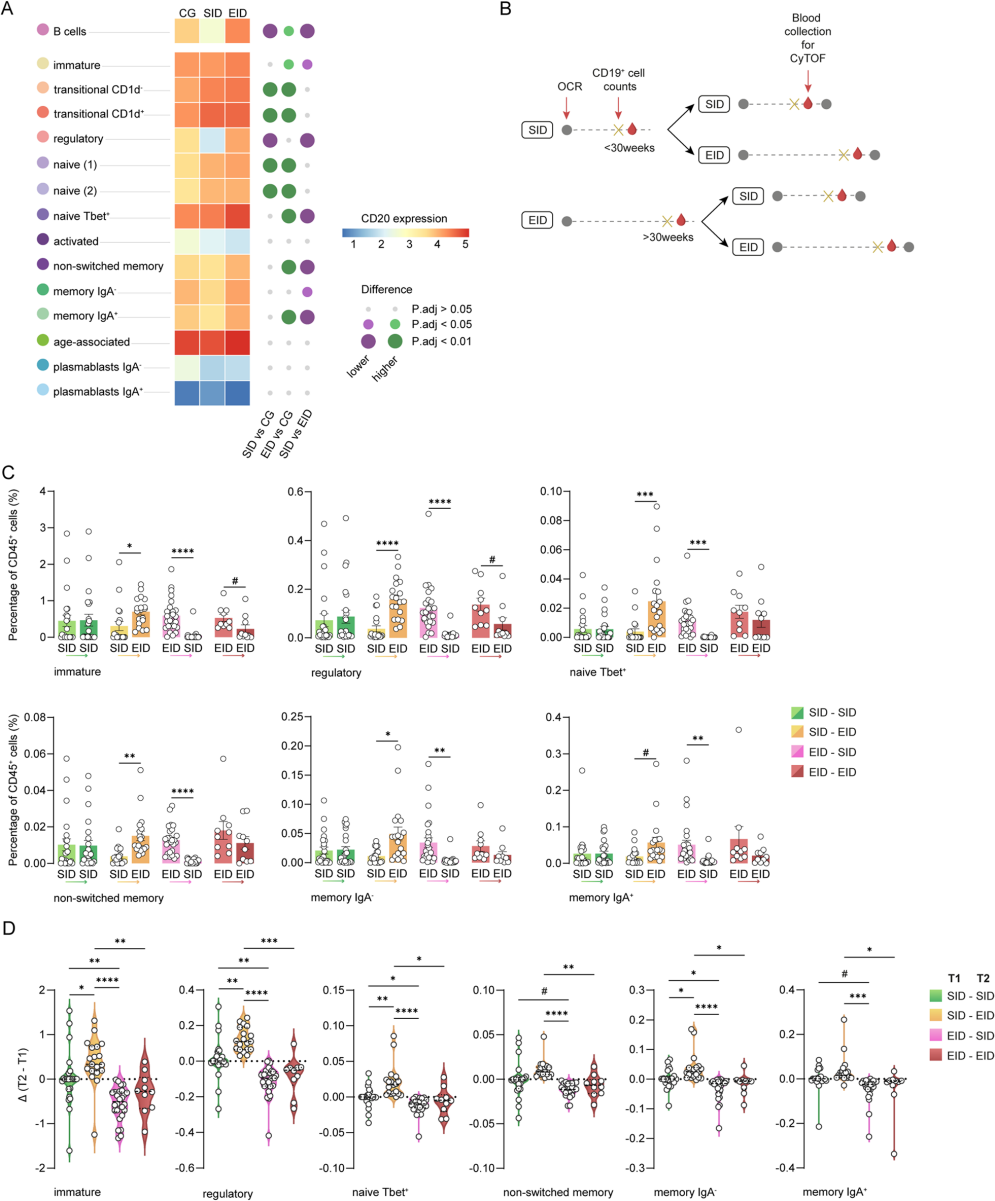
Repopulated B cell subsets increase CD20 expression. **A** Heatmap indicates CD20 median scaled intensity across B cell subpopulations. The size of the circles (right) represents the size of the p-value and the colour represents the direction of change between the different experimental groups. **B** Schematic overview of the longitudinal study design and timeline. **C** Bar plots display the percentage of annotated B cell subsets out of the total CD45^+^ cells from patients that went from SID to SID, SID to EID, EID to SID and EID to EID. Each data point corresponds to each individual, columns and error bars show mean ± SEM. P-values indicate the statistical differences after a GLMM model with age, sex and type of MS as covariates and patient ID as a random effect. **D** Violin plots display the Δ or subtraction of the percentage of annotated B cell subsets out of the total CD45^+^ cells at the second blood sampling minus the percentage of annotated B cell subsets out of the total CD45^+^ cells at the first blood sampling. P-values indicate the statistical differences after a GLM model of the change of percentages between groups of patients, with age, sex and type of MS as covariates. *adjusted p-value < 0.05, **adjusted p-value < 0.01, ***adjusted p-value < 0.001, ****adjusted p-value < 0.0001; #unadjusted p-value < 0.05. *OCR* ocrelizumab, *GLM* multivariate general linear model; *GLMM* multivariate general linear mixed model, *CG* control group, *SID* standard interval dosing, *EID* extended interval dosing, *T1* first-time point/blood sampling, *T2* second-time point/blood sampling

We observed that repopulated B cells in EID patients had an overall higher expression of CD20 at the time of blood collection, i.e. just before the next dose of ocrelizumab, compared to CG and SID patients while SID patients had lower expression of CD20 compared to both CG and EID patients (Fig. 4A; Additional file 2: Table S5). However, when we distinguished between different B cell subsets, we found that repopulated transitional and naïve B cells in SID expressed more CD20 compared to those in CG patients, whereas memory B cells had increased CD20 expression in EID compared to CG patients. As expected, CD20 expression was very low in plasmablasts, explaining the lack of depletion of plasmablasts after ocrelizumab (Fig. 2B). Furthermore, we observed that repopulated immature, regulatory, naïve Tbet^+^ and memory B cell subsets in EID patients had higher CD20 expression than in SID patients (Fig. 4A).

To assess whether the increase in CD20 after extended dosing increases B cell depletion of a consecutive ocrelizumab injection, we analysed a longitudinal second sample taken before the next infusion of ocrelizumab for a subset of patients. Next, we compared B cell repopulation in patients that went from SID to SID, SID to EID, EID to SID and EID to EID (Fig. 4B). Of note, there were no significant differences in the number of patients in these 4 groups. In accordance with increased CD20 expression in repopulated B cells after EID, we observed that the next round of B cell depletion by ocrelizumab was more effective after EID than after SID (Fig. 4C, D; Additional file 1: Fig. S4A, B, Additional file 2: Tables S6, S7). More specifically, we observed a significant reduction in most B cell subsets after the next dose of ocrelizumab, especially in EID to SID patients. In contrast, patients that went from SID to EID had higher levels of repopulating B cells. Furthermore, there was a trend towards less repopulation of most B cell subsets after the second dose in EID to EID patients, whereas there were no clear differences after the first or second dose in SID to SID patients (Fig. 4C, D, Additional file 1: Fig. S4A, B). Lastly, 11 of the non-ocrelizumab treated patients (CG) switched to ocrelizumab as a second-time point and were divided into SID and EID (Additional file 1: Fig. S4C, D, Additional file 2: Table S8). These patients showed similar trends of B cell subsets repopulation as the cross-sectional study (Fig. 2C). However, due to the low number of patients, we could not apply the linear mixed model and no other statistical analysis could be performed. Taken together, treating patients with consecutive EID intervals might lead to a lower extent of repopulation of B cells in the next infusion of ocrelizumab. This could be due to a prolonged depletion of B cells since they have increased CD20 expression.

### B cell repopulation does not correlate with patient characteristics nor disease parameters

**Fig. 5:**
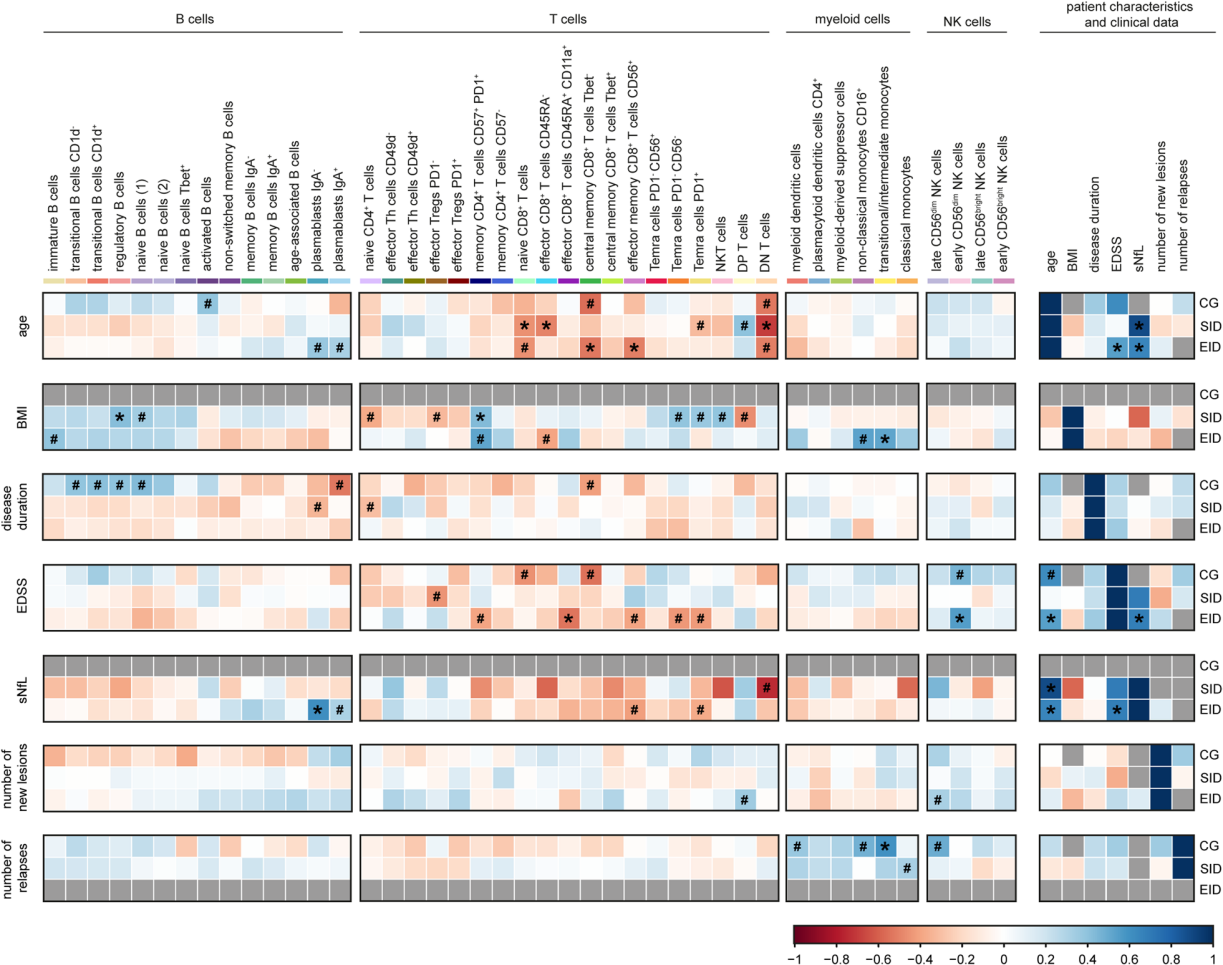
Repopulated B cell subsets do not correlate with the number of relapses nor radiological activity. Heatmap indicates the Spearman correlation values between each annotated immune cell subset and patient characteristics and clinical data. Colour key represents positive (blue) and negative (red) correlation. Grey colour indicates that no correlation was measured, either because less than 10 data point were included or because all values were the same. *adjusted p-value < 0.05, #unadjusted p-value < 0.05. *Th* T helper cells, *Tregs* regulatory T cells, *Temra* T effector memory re-expressing CD45RA cells, *NKT* natural killer T cells, *DN* double negative, *DP* double positive cells, *NK* natural killer cells, *BMI* body mass index, *EDSS* expanded disability status scale, *sNfL* serum neurofilament light, *CG* control group, *SID* standard interval dosing, *EID* extended interval dosing

Next, we assessed if immune cell changes after different dosage intervals differentially correlate with patient characteristics and clinical parameters at timepoint of PBMC collection. For this, we performed Spearman correlations of all immune cell subsets with the age, BMI, disease duration, EDSS, sNfL concentration, number of new lesions and number of relapses since treatment (Fig. 5). Overall, B cell repopulation did not significantly correlate with patient characteristics nor clinical data. However, an increase in regulatory B cells was significantly associated with a higher BMI in SID patients and a similar positive trend was observed in EID. IgA^−^ plasmablasts positively correlated with sNfL levels in EID patients, and similarly, this cell type also had a trend to positively correlate with older age. Interestingly, repopulated B cell subsets did not correlate with the number of new lesions and the number of relapses. Furthermore, EID patients did not have any relapses and therefore, we could not make any correlation.

A few subsets of T cells, such as effector CD8^+^ T cells, positively correlated with age and EDSS, while PD1^−^ CD56^−^ and PD1^+^ Temra cells were associated with a negative trend with BMI. Finally, we also observed a positive correlation between the presence of myeloid cells and the number of relapses in CG. From these correlations and given that SID and EID patients did not differ in relapse rate and (markers of) disease progression, we concluded that ocrelizumab works regardless of the differences in B cell repopulation between SID and EID patients. In both types of dosing intervals, there was less radiological activity and fewer relapses than in CG. Hence, extending ocrelizumab dosing leads to a change in B cell repopulation but there are no effects on the clinical data.

## Discussion

Here, we aimed to study how SID and EID of ocrelizumab affect the peripheral immune cell compartment, with a specific focus on repopulated B cells. We further assessed if extending the dosing of ocrelizumab and the corresponding repopulated B cells influenced the effectiveness of the therapy. Using a CyTOF multi-parameter approach, we now provide a comprehensive overview of the immune cell subsets after treatment with ocrelizumab. We revealed that various B cell subsets are repopulating slightly differently in EID vs. SID patients, but these differences did not correlate with disease parameters, such as the number of relapse rate nor number of lesions. We furthermore show that B cells that are repopulating in both dosing schemes have increased expression of migratory markers, which could indicate a higher migratory capacity towards the CNS. Finally, we observed an increase in CD20 expression on B cells in EID patients, which was associated with a lower repopulation of B cells after consecutive EID dosages.

We show that the first B cell subsets that repopulate after ocrelizumab are immature and transitional B cells, and that this repopulation is slightly different between SID and EID patients. B cells are lymphocytes of the adaptive immune system that originate from the bone marrow and subsequently develop into immature B cells and transitional B cells. These cells will migrate toward the periphery to become mature naïve B cells. After encountering an antigen, naïve B cells will become active and differentiate into short-lived plasma cells or memory B cells in the germinal centers. After the second interaction with an antigen in the germinal centers, B cells proliferate and transform into long-lived memory or plasma cells [24, 25]. Hence, it is expected that after B cell depletion, the first B cells to repopulate are immature, transitional and naïve B cells, which has been shown with different anti-CD20 therapies such as rituximab [26–28]. This was corroborated in our study, although we observed slight differences between SID and EID patients. In particular, there is a slight increase of early B cell subsets, such as immature or transitional B cells, in EID patients compared to SID, despite equal B cell counts two weeks before the PBMC collection. This could suggest that EID patients have faster repopulation or development of early B cells in those two weeks than SID patients.

After rituximab, B cells reappear with a more activated phenotype, shown by an increase of CD25, CD40, CD69 and CD86 [27]. We were wondering if repopulating B cells after ocrelizumab treatment showed an effect on the expression of migratory markers. After ocrelizumab, B cells reappear with increased expression of key migratory markers CD11a, CD49d, CD54 and CD162. This could indicate that B cells have a greater capacity to migrate across the different CNS barriers [22, 29]. Further research is needed to show if an enhanced expression of migratory markers coincides with increased numbers of B cells in the CNS of patients with MS and whether this affects disease progression. We now know that during MS, certain B cells and especially plasma cells can control neuroinflammation by the secretion of anti-inflammatory cytokines, such as IL-35 and IL-10 [30, 31]. Hence, the increased migration capacity of anti-inflammatory B cells into the CNS could be beneficial for the patient. In our cohort, we have defined various possible anti-inflammatory cells, such as regulatory B cells by the expression of CD1d and CD5 as previously defined, and IgA^+^ plasmablasts [30, 32–34]. However, we can only speculate about their function since this regulatory function can only be confirmed by the production of anti-inflammatory cytokines.

CD20 is a protein expressed on the majority of B cell subsets, but its expression can be lost in plasmablasts and plasma cells [35, 36]. Consequently, plasmablasts are commonly less targeted by ocrelizumab as we also see in our cohort [37]. Interestingly, ocrelizumab can also target CD20^+^ T cells since T cells can acquire CD20 via trogocytosis when interacting with antigen-presenting B cells [38–40]. However, following our unbiased clustering approach, we could not detect CD3^+^ CD20^+^ T cells in our cohort. In our case, the developed CyTOF panel was focused on B cells and allowed us to distinguish all B cell differentiation stages and some T and myeloid cell subsets. While we did not find major differences in T cell subset numbers after SID or EID, recent data suggest that ocrelizumab affects effector and memory CD4^+^ and CD8^+^ T cell numbers, but not the diversity of their T cell receptor repertoire [41–43]. Remarkably, we found an increased expression of CD20 on repopulated B cell types, especially after EID. We speculated that this could lead to less repopulation of B cells, maybe due to a stronger or more effective depletion of these cells. While we indeed see that after consecutive EID intervals, there is less B cell repopulation, further research is needed to understand the mechanisms behind this process and its potential implications for ocrelizumab treatment.

One important criterion of personalized dosing is that patients are put into different dosing schemes depending on their B cell counts. Therefore, our conclusions based on these results might be dependent on the individual differences and not per se, the treatment. It could be that EID patients have already intrinsic higher CD20 expression on their B cells, for example. We are currently collecting more samples from patients with MS and their clinical data to understand why some patients require a longer time to repopulate B cells and have to be treated with an EID interval. In our cohort, we show that continuously extending the interval of ocrelizumab might lead to less repopulation of B cells and still maintain the efficacy of the therapy. Interestingly, other studies have also shown that extending the dosing interval of ocrelizumab did not have a detrimental effect on the patients [12, 44–47], except in one study where extending the interval to more than 24 weeks was associated with a higher risk of radiological activity [48]. The study of Baker et al. reporting on extension data of the phase II ocrelizumab trial, showed that despite B cell repopulation after discontinuation of ocrelizumab, disease activity remained low during a 42-week treatment free period. While in our and other studies, a small B cell repopulation could be observed already at 24 weeks, B cell repopulation to the lower limit of normal only occurred around 70–80 weeks after ocrelizumab treatment [12, 46–48]. Further research is needed to assess how different extended intervals affect B cell repopulation. In our cohort, we considered an extended interval of ocrelizumab after 30 weeks, at which point B cell repopulation could already be observed, and, similarly to most previous studies, we did not detect an increase in radiological activity nor an association of the repopulated B cell subsets with it.

Our study has a few limitations, first that our CG group is composed of patients treated with different DMTs. Consequently, the results cannot be extrapolated to all untreated patients with MS, but we consider it still a good control group to study the repopulation of B cells after ocrelizumab since these DMTs have other mechanisms of action [49]. Yet, one patient was treated with alemtuzumab, a potent lymphocyte-depleting agent [9]. While CD19^+^ B cells are largely replenished 3 months after alemtuzumab treatment [9], and we therefore took a blood sample 3 months after the last alemtuzumab infusion, it has been suggested that alemtuzumab induces long-term depletion of memory B cells [9, 50]. However, we did not observe this patient to be an outlier in any of the B cell populations studied here, including memory B cells. Secondly, we have a lack of longitudinal samples, especially for CG patients and subsequently, we could not perform statistics for all the analyses. Thirdly, we plotted our results as percentages within immune cells, which is susceptible to interpretation. However, due to technical and biological limitations of CyTOF data, we find that percentages are more reliable to work with than absolute counts of each cell subset. Finally, one of the main difficulties in CyTOF data is to biologically name the immune cell clusters with the antibodies used. We have tried to biologically annotate the clusters as accurately as possible, but we provide the heatmaps and, especially, the median expression values of these heatmaps for a full overview of the marker expression.

Taken together, we have provided the immune cell landscape after treatment with ocrelizumab. We have shown that B cells repopulate differently after standard or extended intervals of ocrelizumab and that both treatment strategies (EID and SID) are effective. Thus, regardless of the slight differences in B cell repopulation between SID and EID, both intervals seem clinically effective for the patient. We also encourage further studies to confirm the safety and efficacy of extending ocrelizumab dosages in the long term.

### Supplementary Information

**Additional file 1: Figure S1.** Pre-gating strategy of the CyTOF. **A**. Representation of dotplots of the pre-gating strategy of the data obtained with the CyTOF. (1) Removal of cell debris, beads and doublets, (2) cleaning signal over time with flowCut, (3) debarcoding, (4) selection of CD45^+^ live cells and (5) batch normalization with CytoNorm. **B**. Dotplot represents the pre-gating of CD3^+^CD19^−^, CD3^−^CD19^+^ and CD3^−^CD19^−^ cells for further analysis. **Figure S2**. B cell subclustering. **A**. Heatmap displays the median scaled intensities of all the markers across the annotated B cell subclusters. **B**. Bar plots of the percentage of each annotated B cell subpopulation out of the total CD19^+^ cells from CG, SID and EID patients. Each data point corresponds to each individual, columns and error bars show mean ± SEM. P-values indicate the statistical differences after a GLM model with age, sex and type of MS as covariates. *adjusted p-value < 0.05, **adjusted p-value < 0.01, ***adjusted p-value < 0.001, ****adjusted p-value < 0.0001. GLM = multivariate general linear model; CG = control group; SID = standard interval dosing; EID = extended interval dosing. **Figure S3**. T cell and rest of immune cell subclustering. **A**. Heatmap shows the median scaled intensities of all the markers across the annotated T cell subclusters. **B**. Heatmap represents the median scaled intensities of all the markers across the annotated CD3^−^CD19^−^ cell subclusters. Th = T helper cells; Tregs = regulatory T cells; Temra = T effector memory re-expressing CD45RA cells; NKT = natural killer T cells; DN = double negative; DP = double positive cells; NK = natural killer cells. **Figure S4**. Longitudinal cohort after treatment with standard or extended interval dosing of ocrelizumab. **A**. Bar plots display the percentage of annotated B cell subsets out of the total CD45 + cells from patients that went from SID to SID, SID to EID, EID to SID and EID to EID. **B**. Violin plots display the Δ or subtraction of the percentage of annotated B cell subsets out of the total CD45^+^ cells at the second blood sampling minus the percentage of annotated B cell subsets out of the total CD45^+^ cells at the first blood sampling. P-values indicate the statistical differences after a GLM model of the change of percentages between groups of patients, with age, sex and type of MS as covariates. **C**. Schematic overview of the longitudinal study design and timeline for CG patients. **D**. Percentage of annotated B cell subsets out of the total amount of CD45^+^ cells from patients that went from CG to SID and CG to EID. **A and D**. Each data point corresponds to each individual, columns and error bars show mean ± SEM. P-values indicate the statistical differences after a GLMM model with age, sex and type of MS as covariates and patient ID as a random effect. *adjusted p-value < 0.05, **adjusted p-value < 0.01, ***adjusted p-value < 0.001, ****adjusted p-value < 0.0001; #unadjusted p-value < 0.05. OCR = ocrelizumab; GLM = multivariate general linear model; GLMM = multivariate general linear mixed model; CG = control group; SID = standard interval dosing; EID = extended interval dosing; T1 = first-time point/blood sampling; T2 = second-time point/blood sampling.

**Additional file 2: Table S1.** Median expression of markers. **Table S2.** Mean and standard deviation of the percentage of each immune cell subset. **Table S3.** Mean and standard deviation of the percentage of each B cell subset among all CD19^+^ cells. **Table S4.** Median expression markers of migratory markers. **Table S5.** Median expression markers of migratory markers. **Table S6.** Mean and standard deviation of the percentage of each B cell subset among all CD45^+^ cells with follow-up sample. **Table S7.** Statistics of Δ. **Table S8.** Mean and standard deviation of the percentage of each B cell subset among all CD45^+^ cells with follow-up sample in GC patients.

## Data Availability

Anonymized data, not published in the article, will be shared upon reasonable request from a qualified investigator.
