# Supplementary material for: Extended interval dosing of ocrelizumab modifies the repopulation of B cells without altering the clinical efficacy in multiple sclerosis
Source: J Neuroinflammation. 2023 Sep 26;20:215. doi: 10.1186/s12974-023-02900-z (PMC10521424; doi:10.1186/s12974-023-02900-z)
Supplement: Supplementary file 1 — Additional file 1: Figure S1. Pre-gating strategy of the CyTOF. A. Representation of dotplots of the pre-gating strategy of the data obtained with the CyTOF. (1) Removal of cell debris, beads and doublets, (2) cleaning signal over time with flowCut, (3) debarcoding, (4) selection of CD45+ live cells and (5) batch normalization with CytoNorm. B. Dotplot represents the pre-gating of CD3+CD19−, CD3−CD19+ and CD3−CD19− cells for further analysis. Figure S2. B cell subclustering. A. Heatmap displays the median scaled intensities of all the markers across the annotated B cell subclusters. B. Bar plots of the percentage of each annotated B cell subpopulation out of the total CD19+ cells from CG, SID and EID patients. Each data point corresponds to each individual, columns and error bars show mean ± SEM. P-values indicate the statistical differences after a GLM model with age, sex and type of MS as covariates. *adjusted p-value < 0.05, **adjusted p-value < 0.01, ***adjusted p-value < 0.001, ****adjusted p-value < 0.0001. GLM = multivariate general linear model; CG = control group; SID = standard interval dosing; EID = extended interval dosing. Figure S3. T cell and rest of immune cell subclustering. A. Heatmap shows the median scaled intensities of all the markers across the annotated T cell subclusters. B. Heatmap represents the median scaled intensities of all the markers across the annotated CD3−CD19− cell subclusters. Th = T helper cells; Tregs = regulatory T cells; Temra = T effector memory re-expressing CD45RA cells; NKT = natural killer T cells; DN = double negative; DP = double positive cells; NK = natural killer cells. Figure S4. Longitudinal cohort after treatment with standard or extended interval dosing of ocrelizumab. A. Bar plots display the percentage of annotated B cell subsets out of the total CD45 + cells from patients that went from SID to SID, SID to EID, EID to SID and EID to EID. B. Violin plots display the Δ or subtraction of the percentage of annotat [file 12974_2023_2900_MOESM1_ESM.docx]

**Extended interval dosing of ocrelizumab modifies the repopulation of B cells without altering the clinical efficacy in multiple sclerosis**

Carla Rodriguez-Mogeda^1,2,3^, Zoë Y.G.J. van Lierop^2,3,4^, Susanne van der Pol^1,2,3^, Loet Coenen^1,2,5^, Laura Hogenboom^2,3,4^, Alwin Kamermans^1,2,3^, Ernesto Rodriguez^1,6,7^, Jack van Horssen^1,2,3^, Zoé L.E. van Kempen^2,3,4^, Bernard M.J. Uitdehaag^2,3,4^, Charlotte E. Teunissen^2,8,9^, Maarten E. Witte^1,2,3,7^, Joep Killestein^2,3,4^, Helga E. de Vries^1,2,3^

**Additional File 1: Supplementary Figures**

**
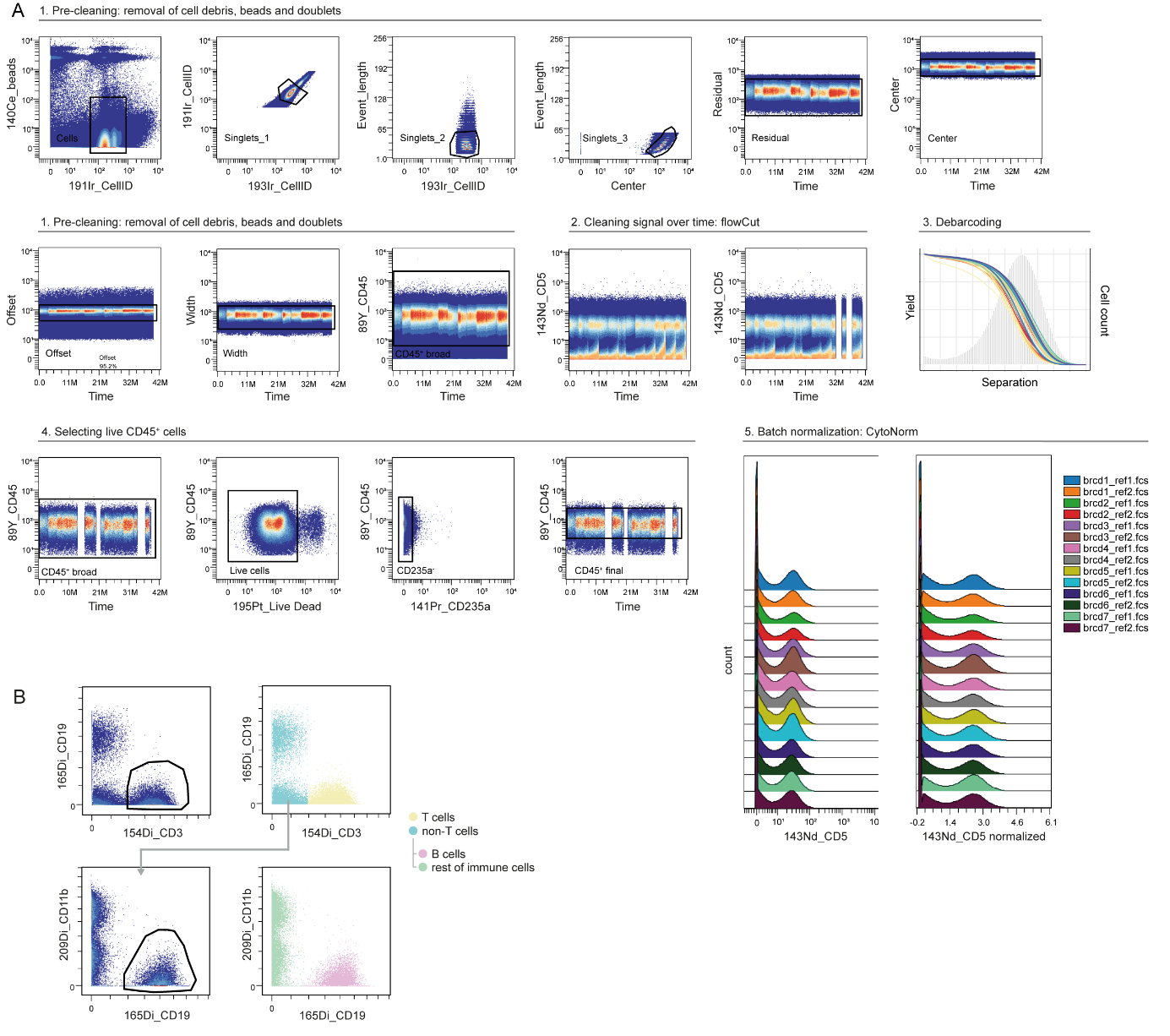
**

**Figure S1.** Pre-gating strategy of the CyTOF.

**A**. Representation of dotplots of the pre-gating strategy of the data obtained with the CyTOF. (1) Removal of cell debris, beads and doublets, (2) cleaning signal over time with flowCut, (3) debarcoding, (4) selection of CD45^+^ live cells and (5) batch normalization with CytoNorm. **B**. Dotplot represents the pre-gating of CD3^+^CD19^-^, CD3^-^CD19^+^ and CD3^-^CD19^-^ cells for further analysis.


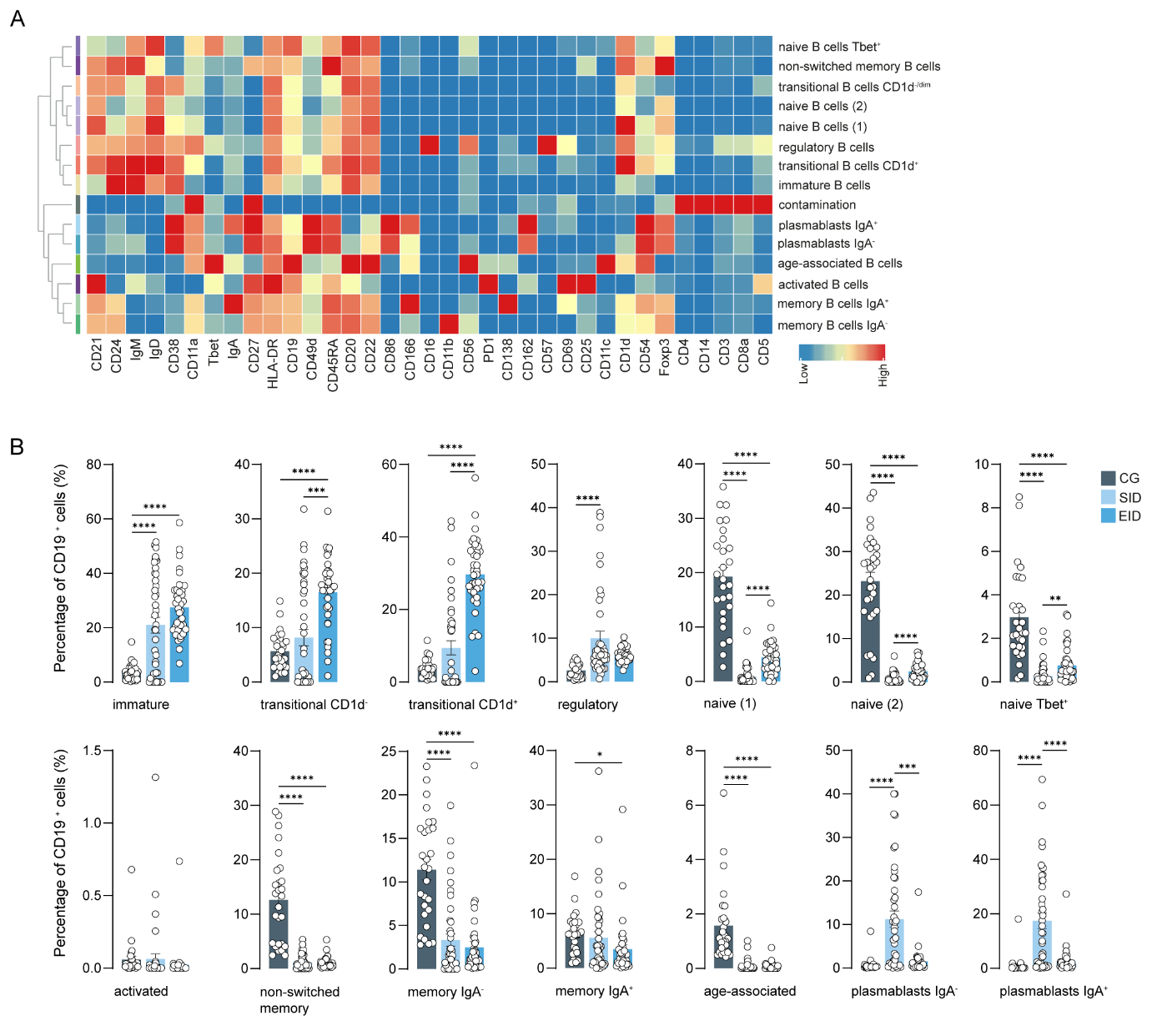


**Figure S2**. B cell subclustering.

**A**. Heatmap displays the median scaled intensities of all the markers across the annotated B cell subclusters. **B**. Bar plots of the percentage of each annotated B cell subpopulation out of the total CD19^+^ cells from CG, SID and EID patients. Each data point corresponds to each individual, columns and error bars show mean ± SEM. P-values indicate the statistical differences after a GLM model with age, sex and type of MS as covariates. *adjusted p-value < 0.05, **adjusted p-value < 0.01, ***adjusted p-value < 0.001, ****adjusted p-value < 0.0001. GLM = multivariate general linear model; CG = control group; SID = standard interval dosing; EID = extended interval dosing.


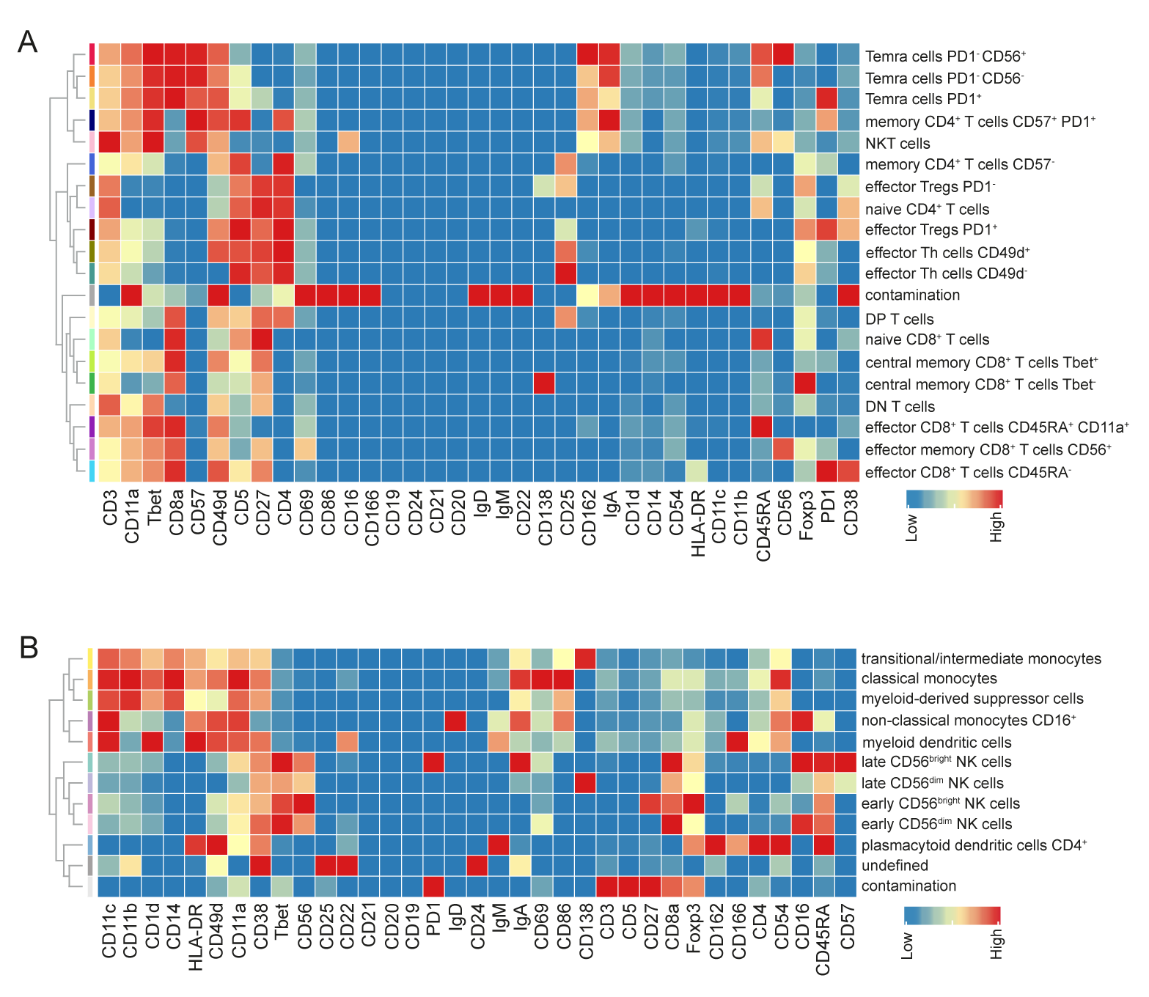


**Figure S3**. T cell and rest of immune cell subclustering.

**A**. Heatmap shows the median scaled intensities of all the markers across the annotated T cell subclusters. **B**. Heatmap represents the median scaled intensities of all the markers across the annotated CD3^-^CD19^-^ cell subclusters. Th = T helper cells; Tregs = regulatory T cells; Temra = T effector memory re-expressing CD45RA cells; NKT = natural killer T cells; DN = double negative; DP = double positive cells; NK = natural killer cells.


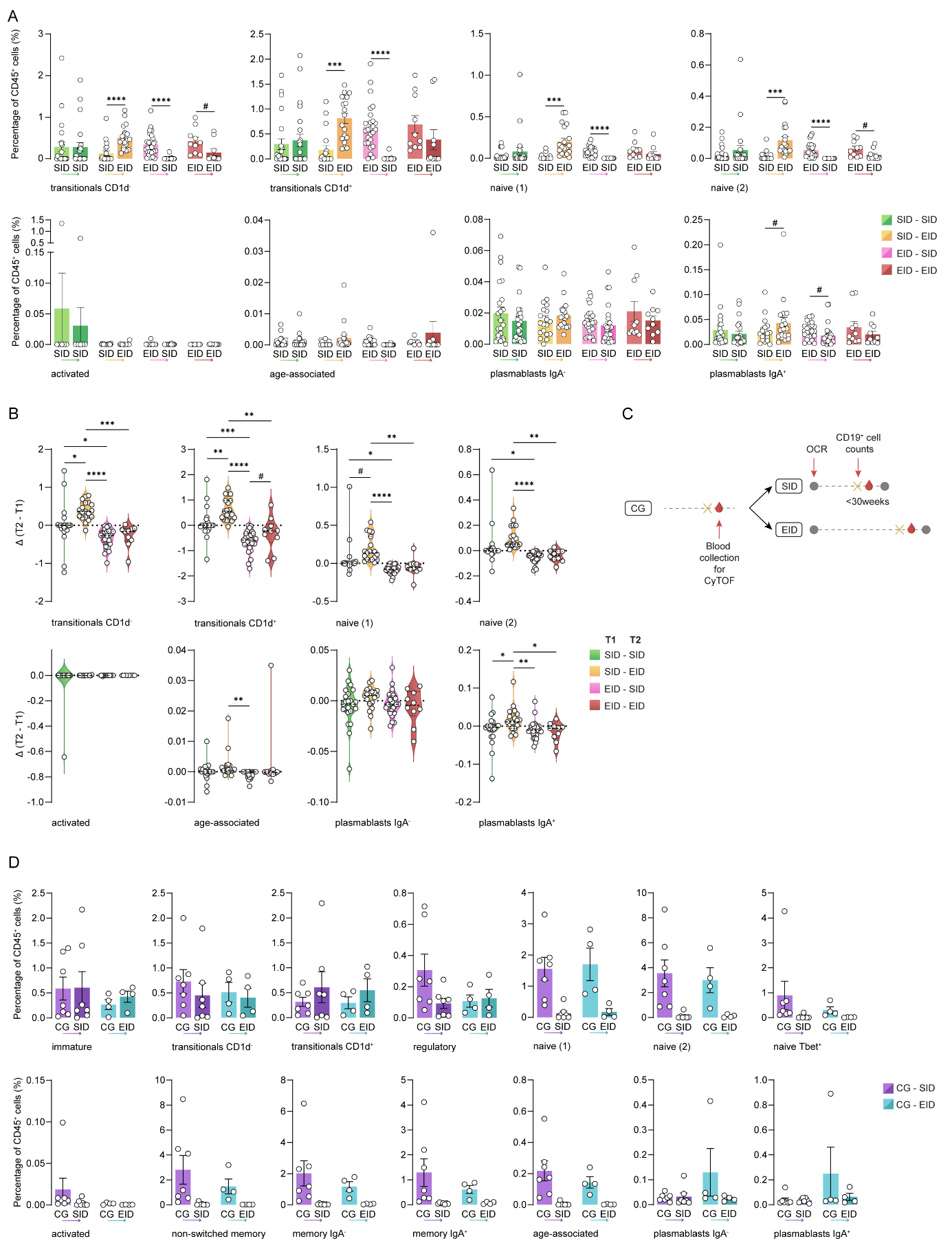


**Figure S4**. Longitudinal cohort after treatment with standard or extended interval dosing of ocrelizumab.

**A**. Bar plots display the percentage of annotated B cell subsets out of the total CD45+ cells from patients that went from SID to SID, SID to EID, EID to SID and EID to EID. **B**. Violin plots display the Δ or subtraction of the percentage of annotated B cell subsets out of the total CD45^+^ cells at the second blood sampling minus the percentage of annotated B cell subsets out of the total CD45^+^ cells at the first blood sampling. P-values indicate the statistical differences after a GLM model of the change of percentages between groups of patients, with age, sex and type of MS as covariates. **C**. Schematic overview of the longitudinal study design and timeline for CG patients. **D**. Percentage of annotated B cell subsets out of the total amount of CD45^+^ cells from patients that went from CG to SID and CG to EID. **A and D**. Each data point corresponds to each individual, columns and error bars show mean ± SEM. P-values indicate the statistical differences after a GLMM model with age, sex and type of MS as covariates and patient ID as a random effect. *adjusted p-value < 0.05, **adjusted p-value < 0.01, ***adjusted p-value < 0.001, ****adjusted p-value < 0.0001; #unadjusted p-value < 0.05. OCR = ocrelizumab; GLM = multivariate general linear model; GLMM = multivariate general linear mixed model; CG = control group; SID = standard interval dosing; EID = extended interval dosing; T1 = first-time point/blood sampling; T2 = second-time point/blood sampling.
